# Tensile Properties and Fracture Behaviour of Biodegradable Iron–Manganese Scaffolds Produced by Powder Sintering

**DOI:** 10.3390/ma12101572

**Published:** 2019-05-14

**Authors:** A. Dehghan-Manshadi, D.H. StJohn, M.S. Dargusch

**Affiliations:** Queensland Centre for Advanced Materials Processing and Manufacturing (AMPAM), School of Mechanical and Mining Engineering, The University of Queensland, St Lucia, QLD 4072, Australia; a.dehghanmanshadi@uq.edu.au (A.D.-M.); d.stjohn@uq.edu.au (D.H.S.)

**Keywords:** Fe–Mn alloys, biodegradable materials, mechanical properties, powder sintering

## Abstract

Powder sintering at 1200 °C for 180 min was used to produce Fe–Mn based alloys with tensile properties and an elastic modulus suitable for biodegradable implant applications. The effect of the addition of manganese on the microstructure, tensile properties and fracture behaviour of the Fe–Mn alloys was investigated. The Fe–35Mn alloy with a microstructure dominated by the Austenite phase showed the best set of tensile properties, including ultimate tensile strength and Young’s modulus, suitable for orthopaedic implant applications. The fracture surface of the Fe–35Mn alloy showed signs of complex multimode fracture behaviour, consisting of interconnected pores and large segments with signs of ductile fracture, including the presence of dimples as well as micro-voids.

## 1. Introduction

The development of metallic materials for surgical implants has been the focus of a significant number of research activities in recent years. Ideal materials for implants should have excellent biocompatibility, a porous structure, biodegradability and adequate mechanical properties that are close to those of actual tissue in the human body. While traditional biomedical metallic materials, such as stainless steel, titanium (Ti) and cobalt (Co) alloys have excellent biocompatibility and mechanical properties [1,2,3,4,5,6], they have no biodegradability, and will therefore remain in the body as a foreign object, risking the possibility of long-term negative effects. Therefore, the development of biodegradable implants provides an alternative to implants fabricated from Ti, Co and stainless steel. 

Very few metallic and non-metallic biodegradable implants have been commercially available. Synthetic biodegradable polymers with good biocompatibility and degradability are the most common materials commercially available [7,8]. The advantage of these polymer materials is that the degradability rate can be tailored by molecular weight design [9]. However, these materials suffer from poor mechanical properties, which challenges their suitability for hard tissue engineering, including applications such as scaffolds for bone replacement. 

On the other hand, metals are historically known as materials with adequate strength, toughness and formability, all of which are essential for hard tissue substitution engineering. Many metal alloys also have an acceptable biocompatibility and biodegradability rate, which makes them attractive materials for biomedical applications, with superior strength compared to their synthetic polymer counterparts. Magnesium (Mg), Iron (Fe), Tungsten (W) and Zinc (Zn) are among the metallic material systems that have attractive properties for biomedical implants, and these materials have already found some applications as stents and hard tissue implants, as well as scaffolds for bone surgery [10,11]. 

Mg-based materials [12] are currently the most common biodegradable metallic materials, but the degradation rate of magnesium alloys may be too rapid for some implant applications, and under some circumstances the device may lose its mechanical/functional integrity within the patient’s body [13,14]. Additionally, the fast degradation of Mg within the body may produce by-products (i.e., Mg ions and micron size particles) at a rate faster than what the body can manage to incorporate. Another important issue with Mg as a biomaterial is the release of hydrogen gas during its degradation process, which may cause the accumulation of large amounts of hydrogen as subcutaneous gas bubbles [15]. While the aforementioned problems with bio-application of Mg have been improved with different strategies such as alloying [10,15,16,17], surface treatment [18,19] and coating [20,21,22], magnesium and its alloys are still far from an ideal biodegradable implant material. 

Compared with Mg-based materials, Fe-based materials have superior mechanical properties, and are more attractive particularly from a structural perspective. Fe is even more biocompatible than Mg, and is known as an essential element that plays significant roles in human body metabolism, including in the transport, activation, and storage of molecular oxygen [23]. However, the degradation rate of pure Fe is slow, and preliminary animal tests have also revealed a slow in vivo degradation rate [10]. As a result of such a slow degradation rate, it has been reported that large portions of the pure Fe stent remained intact in blood vessels even twelve months after implantation [24]. Another big issue with iron-based implants relates to the ferromagnetic nature of Fe, which causes incompatibility with magnetic resonance imaging (MRI). Much research has been undertaken to improve the biocompatibility of iron by adopting strategies to promote a faster degradation rate and improved MRI compatibility. Alloying, microstructure modification, diverse manufacturing processes and surface treatment are among the techniques that have assisted in the improvement of the biocompatibility of iron [11,25,26]. 

Among all the steps that have been adopted to improve the biocompatibility and biodegradability of Fe, alloying with manganese (Mn) seems a promising solution [25,27]. For instance, alloying with Mn (at fractions over 29 wt. %) provides anti-magnetism behaviour, due to its complete austenitic structure. Mn also increases the corrosion (degradation) rate of iron [26]. On the other hand, Mn is a trace element that is necessary in many enzymatic reactions [10], and has no/fewer toxicity effects. 

Hermawan et al. [25,28,29] performed a comprehensive study on manufacturing and biocompatibility of a wide range of dense Fe–Mn alloys. They found that the Fe–35Mn alloy has good mechanical, magnetic and corrosion properties, as required for application as biodegradable stents. However, the potential application of dense Fe–Mn alloys may be suitable for stents, but is not suitable for implants and scaffolds for application in bone surgery, where a degree of porosity is essential. Integration of residual pores within the Fe–Mn structure not only expands its application, but also improves its biodegradability by increasing the exposure area of the material to the corrosive body environment. In this regards, Zhang and Cao [30] employed powder metallurgy and a space holder technique to produce a Fe–35Mn alloy with different porosity levels. However, their product exhibited very poor mechanical properties and was not suitable for implants. Therefore, any attempt to develop porous Fe–Mn alloys with mechanical properties comparable with that of human bone will increase its application range in biomedical engineering.

The aim of the current work was to produce porous Fe–Mn alloys with acceptable mechanical properties. In this regard, three different alloys were prepared with powder metallurgy techniques, and their mechanical properties, microstructure and constituent phases were analysed.

## 2. Materials and Methods

**Figure 1:**
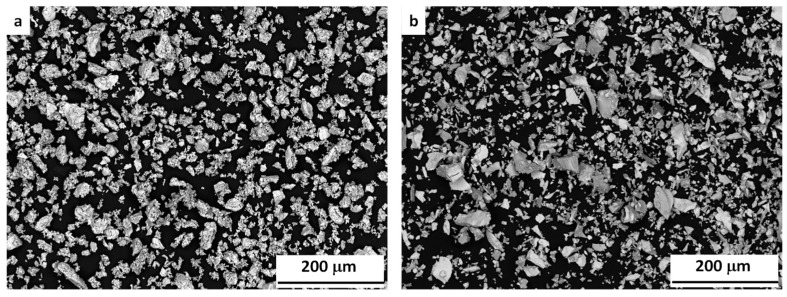
SEM micrograph of initial (**a**) Fe and (**b**) Mn powders.

Three different iron–manganese mixtures, namely Fe–20Mn, Fe–30Mn and Fe–35Mn were prepared by mixing high purity elemental powders of Fe (99% purity, <45 µm, Alfa Aesar, Lancashire, UK) and, Mn (99.5 % purity, <45 µm, Sigma Aldrich, St. Louis, MO, USA). Powder morphologies are shown in Figure 1. The mixing process has been described in detail elsewhere [27]. 

**Figure 2:**
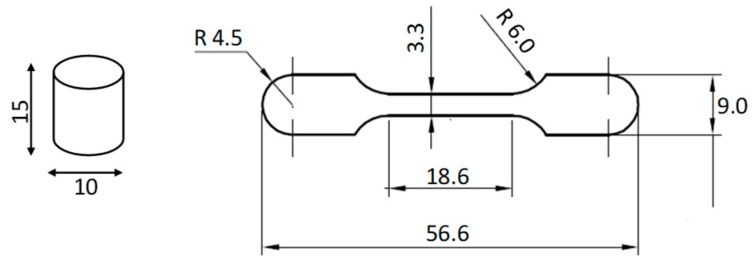
Schematic figure of cylindrical (for microstructure and density studies) and tensile test samples. (Sizes are in mm.)

**Figure 3:**
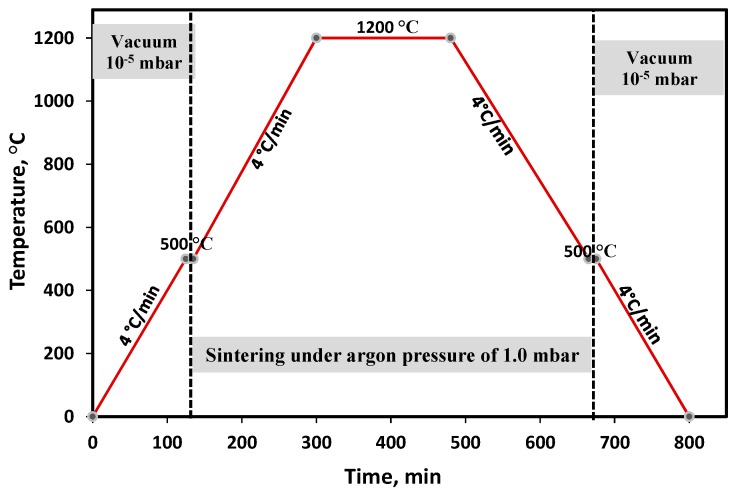
Schematic diagram of the sintering processes.

The mixed powders were pressed in either a floating cylindrical die with a diameter of 10 mm or a tensile ‘dog bone’ die to a maximum pressure of 750 MPa. Figure 2 shows the schematic diagram of the pressed samples. The sintering process was performed at 1200 °C in a high vacuum tube furnace for 180 min with a positive pressure of ~1.0 mbar, using ultra-high purity argon gas. Figure 3 schematically shows the detailed experimental conditions for the sintering process. 

The density of the sintered samples was measured using the Archimedes method (the theoretical densities of Fe and Mn were taken as 7.87 g/cm^3^ and 7.43 g/cm^3^, respectively), and H-Galden ZT-180 (Solvey, Milan, Italy) was used as the fluid. Tensile and compression tests were also performed using an Instron 5584 machine (Instron Inc., High Wycombe, UK) under a strain rate of 3 × 10^−3^ mm/min. A Hitachi TM3030 table-top SEM (Hitachi, Tokyo, Japan) was used for characterisation of powders, sintered structures (Etchant 2.0% Nital) and fracture surfaces. Phases present in the alloys were evaluated using X-ray diffraction (XRD, Bruker, Hamburg, Germany) using the Bruker D8 Advance MKII XRD diffractometer operated at 40 kV and 40 mA with Cu Kα radiation (λ = 0.15406 nm), and diffraction data was analysed using HighScore software. 

## 3. Results and Discussion

### 3.1. Sintered Structures

**Figure 4:**
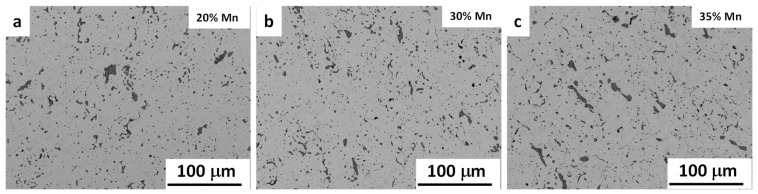
General microstructure of Fe–Mn samples sintered at 1200 °C for 180 min. (**a**) 20% Mn, (**b**) 30% Mn and (**c**) 35% Mn.

The density of the sintered alloys is presented in Table 1 along with calculated porosity values. Significant levels of porosity were observed in the samples as a result of the sintering process. This porosity is a desirable microstructural feature for biodegradable metal implants, particularly as it often appears as open pores that can facilitate both bio integration and bio degradation of the alloys. Although porous structures may be favourable for assimilation, suitable mechanical properties, particularly elongation, must still be maintained. Therefore, further examination of the microstructure, including size and distribution of pores within the sintered samples, was essential. Figure 4 shows the general microstructure of the sintered samples for all three Fe–Mn alloys. Both significant levels of porosity and inclusions (MnO particles) can be observed in the microstructure, with most pores distributed evenly throughout the microstructure with a few larger pore exceptions. 

**Figure 5:**
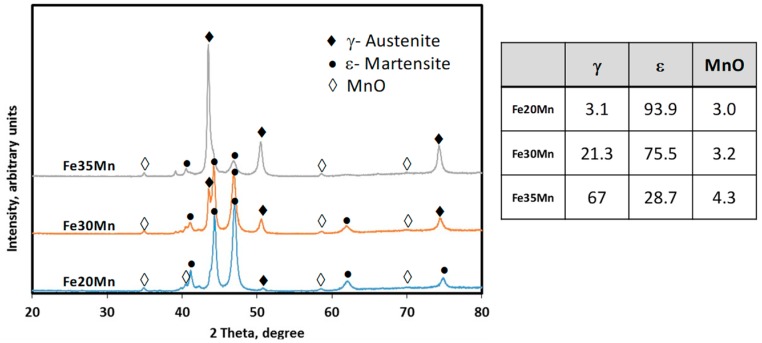
X-ray diffraction (XRD) spectrum of sintered Fe–Mn samples.

To identify the phase composition of each sample, X-ray diffraction (XRD) analysis was undertaken, and the results are summarised in Figure 5. The XRD results show that the phase composition of the alloys changed with manganese levels. While the martensite phase was dominant in the Fe–20Mn sample, as manganese levels were, increased peaks associated with the both austenite (γ) phase and martensite (ε) could be identified until the austenite phase became the dominant phase in the alloy with 35% manganese. The presence of mainly the austenite phase in the Fe–35Mn sample may improve the suitability of the alloy for implant applications, due to the anti-ferromagnetism properties of austenite [27,28]. Between 3–4% of the MnO phase was also present in all samples, which formed during the powder mixing and sintering processes.

### 3.2. Mechanical Properties

**Figure 6:**
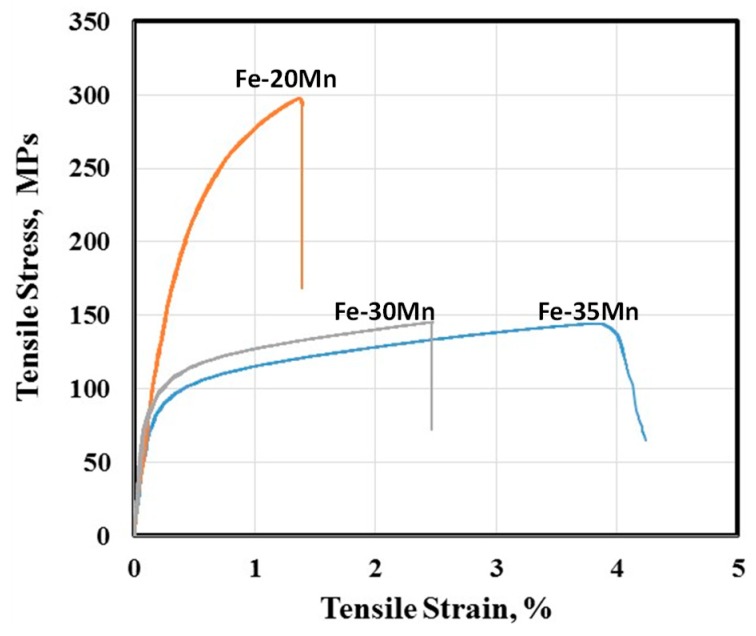
Tensile stress–strain curves of Fe–Mn samples sintered at 1200 °C for 180 min.

Appropriate mechanical properties are essential for the application of biomedical alloys, particularly in load bearing applications such as bone scaffolds, and should be comparable with the properties of natural bone. Figure 6 shows curves obtained from tensile testing of the samples evaluated in this study. All the mechanical properties, including hardness, yield strength, ultimate tensile strength, tensile elongation and Young’s modulus, are also represented in Table 2. In this table, the comparable properties of cortical human bone have also been included [31]. The mechanical properties given in this table indicate that Fe–20Mn had the highest UTS (208 MPa) and lowest elongation (1.5%). Further additions of manganese decreased strength but increased ductility, with the elongation of the Fe–35%Mn alloy exceeding 4% despite porosity levels in this alloy, which exceeded 20%. In summary, the low elongation of the Fe–20Mn and Fe–30Mn alloys were not acceptable for bone substitution applications, but the reasonable elongation values, coupled with good strength and an appropriate modulus, suggest that the alloy containing 35% Mn may be a suitable candidate. A comparison of the results in Table 1 and Table 2 with results reported in the literature shows that the manufacturing process had a significant effect on properties. The sintered samples produced in this investigation had better mechanical properties compared to the results for press and sintered samples reported by Zhang and Cao [30], with their alloys showing much lower strength. However, when cold rolling was performed after powder sintering of Fe–Mn alloys, superior mechanical properties compared to those obtained in the current work were observed and reported [25]. Nonetheless, the biggest advantages of samples produced in the current work is the closer match between their mechanical properties and those of natural bone, as well as the presence of a high fraction of residual and open pores, which are necessary for tissue regeneration after surgery. Additionally, the porous nature of the samples in the current work has the potential to increase the degradation rate of the materials. 

In order to further evaluate the link between microstructure and mechanical properties in the current work, the fracture surfaces were analysed using SEM.

### 3.3. Fracture Surfaces

**Figure 7:**
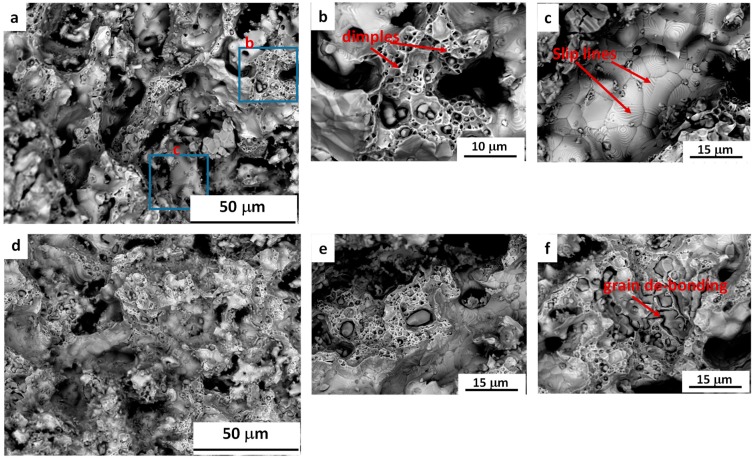
The fracture surfaces of (**a–c**) Fe–35Mn and (**d–f**) Fe–20Mn.

The fracture surface of Fe–20Mn and Fe–35Mn tensile samples are shown in Figure 7. The overall fracture mode is a mixture of ductile and brittle. As illustrated in Figure 7a, the fracture surface of the Fe–35Mn alloy consisted of interconnected pores and large segments of fracture surface, which showed signs of ductile fracture, with the presence of dimples as well as micro-voids (Figure 7b). Additionally, in many areas the fracture surface showed well bonded grains with slip lines visible inside them (Figure 7c). However, this fracture surface also included signs of brittle fracture (i.e., with cleavage features and striations) in some areas. 

In comparison, the fracture surface of the Fe–20Mn sample showed a lower amount of ductile features, but more brittle fracture signs and more isolated pores (Figure 7d–f). Additionally, as shown in Figure 6f, some areas of the fracture surface in this sample demonstrated signs of de-bonding between grains, which is indicative of brittle fracture.

## 4. Conclusions

The microstructure, tensile properties and facture behaviour of a series of Fe–20Mn, Fe–30Mn and Fe–35Mn alloys were investigated. The Fe–35Mn alloy showed the best combination of constituent phases, tensile strength, toughness and Young’s modulus, compared to the target properties of human cortical bone. Examination of the fracture surfaces of the Fe–Mn alloys showed features associated with both ductile and brittle fracture, with the overall fracture mode a mixture of both. The signs of brittle fracture dominated the fracture surface of the Fe–20Mn sample. The fracture surface of the Fe–35Mn alloy showed more signs of ductile fracture, with the presence of dimples as well as micro-voids, but also local areas containing brittle fracture features such as cleavage and striations. This investigation has shown that powder metallurgy can be used to manufacture an Fe–35Mn alloy that has promising properties, including 21 vol. % porosity, 4.0% elongation, a UTS of 144 MPa and a Young’s modulus of 53 GPa, all of which are promising for the application of these materials in biodegradable implants.

## Figures and Tables

**Table 1:** Density of Fe–Mn samples sintered at 1200 °C for 180 min.

Sample	Sintered Density (g/cm^3^)	Sintered Density (%)	General Porosity (%)	Open Porosity (%)
Fe–20Mn	6.70	86.1	13.9	7.8
Fe–30Mn	6.15	79.5	20.6	17.6
Fe–35Mn	6.08	78.7	21.3	53

**Table 2:** Mechanical properties of Fe–Mn samples sintered at 1200 °C for 180 min.

Sample	Hardness (Hv)	Yield Tensile Strength (MPa)	Ultimate Tensile Strength (MPa)	Tensile Elongation (%)	Young Modulus (GPa)
Fe–20mn	162	198	294	1.5	56.6
Fe–30Mn	78	102	145	2.5	55.2
Fe–35Mn	66	94	144	4	53.3
Cortical Human Bone [30]	---	104–121	86–151	1–3	14–17
